# Opinions on Vaccination

**Published:** 1897-08

**Authors:** 


### OPINIONS ON VACCINATION.

Niemeyer says: "It cannot be denied that vaccination sometimes endangers life, and, in some cases, causes cutaneous diseases and scrofulous affections."

Dr. Chas. Creighton, the well-known English pathologist, author of articles on pathology and vaccination, in *The Encyclopedia Brittanica* says the real analogue of cow-pox is not small-pox but syphilis.

